# Next-generation whole exome sequencing to delineate the genetic basis of primary congenital glaucoma

**DOI:** 10.1038/s41598-022-20939-5

**Published:** 2022-10-14

**Authors:** Bushra Rauf, Shahid Y. Khan, Xiaodong Jiao, Bushra Irum, Ramla Ashfaq, Mubashra Zehra, Asma A. Khan, Muhammad Asif Naeem, Mohsin Shahzad, Sheikh Riazuddin, J. Fielding Hejtmancik, S. Amer Riazuddin

**Affiliations:** 1 The Wilmer Eye Institute, Johns Hopkins University School of Medicine, 600 N. Wolfe Street, Maumenee 809, Baltimore, MD 21287 USA; 2 National Centre of Excellence in Molecular Biology, University of the Punjab, Lahore, 53700 Pakistan; 3 Ophthalmic Genetics and Visual Function Branch, National Eye Institute, National Institutes of Health, Bethesda, MD 20892 USA; 4 Jinnah Burn and Reconstructive Surgery Centre, Allama Iqbal Medical College, University of Health Sciences, Lahore, 54550 Pakistan

**Keywords:** Genetics, Eye diseases

## Abstract

To delineate the genetic bases of primary congenital glaucoma (PCG), we ascertained a large cohort consisting of 48 consanguineous families. Of these, we previously reported 26 families with mutations in *CYP1B1* and six families with *LTBP2*, whereas the genetic bases responsible for PCG in 16 families remained elusive. We employed next-generation whole exome sequencing to delineate the genetic basis of PCG in four of these 16 familial cases. Exclusion of linkage to reported PCG loci was established followed by next-generation whole exome sequencing, which was performed on 10 affected individuals manifesting cardinal systems of PCG belonging to four unresolved families along with four control samples consisting of genomic DNAs of individuals harboring mutations in *CYP1B1* and *LTBP2*. The analyses of sequencing datasets failed to identify potential causal alleles in the 10 exomes whereas c.1169G > A (p. Arg390His) in *CYP1B1* and c.3427delC (p.Gln1143Argfs*35) in *LTBP2* were identified in the control samples. Taken together, next-generation whole exome sequencing failed to delineate the genetic basis of PCG in familial cases excluded from mutations in *CYP1B1* and *LTBP2*. These data strengthen the notion that compound heterozygous coding variants or non-coding variants might contribute to PCG.

## Introduction

Glaucoma is the second leading cause of blindness affecting nearly 65 million people worldwide^1,2^. Primary congenital glaucoma (PCG: OMIM # 231300) is a rare form of glaucoma, characterized by defective development of the anterior chamber structures that lead to aqueous outflow obstruction, increased IOP, and optic nerve damage^3,4^. It is usually inherited as an autosomal recessive disorder with incomplete penetrance^3,4^. Increased IOP results in enlargement of the globe (buphthalmos) and irritation of the cornea cause corneal edema/haze. Other clinical findings include Haab’s striae, conjunctival erythema, and optic atrophy in the later stages of the disease^5,6^.

PCG is a genetically heterogeneous disorder and to date, four genetic loci, GLC3A (*CYP1B1*, 2p22-p21), GLC3B (1p36.2–36.1), GLC3C (14q24.3), and GLC3D (*LTBP2*, 14q24.2–24.3) have been reported^7–10^. Mutations in Cytochrome P450 Family 1 Subfamily B Member 1 (*CYP1B1*) (OMIM # 601771) and Latent Transforming Growth Factor-beta Binding Protein 2 (*LTBP2)* (OMIM # 602091) have been identified in patients with autosomal recessive PCG^11,12^, whereas the genes for remaining two genetic loci; GLC3B and GLC3C are yet to be cloned. Moreover, genetic variants responsible for autosomal dominant PCG have been reported^13,14^. Souma and colleagues^13^, reported multiple heterozygous mutations in tunica interna endothelial cell kinase (*TEK*), responsible for autosomal dominant PCG in a multiethnic cohort of familial and sporadic cases. Moreover, Thomson and colleagues^14^, reported one missense and two nonsense heterozygous variants in angiopoietin-1 (*ANGPT1*) responsible for autosomal dominant PCG in three human subjects.

Multiple studies have reported mutations in *CYP1B1* and *LTBP2* responsible for PCG in the Pakistani population^15–19^. We previously reported the identification of pathogenic mutations in *CYP1B1* and *LTBP2* responsible for PCG in families of Pakistani descent^20–22^. Here, we employed next-generation whole exome sequencing to identify the genetic basis of PCG in ten affected individuals belonging to four familial cases excluded for mutations in *CYP1B1* and *LTBP2*.

## Results and Discussion

**Figure 1:**
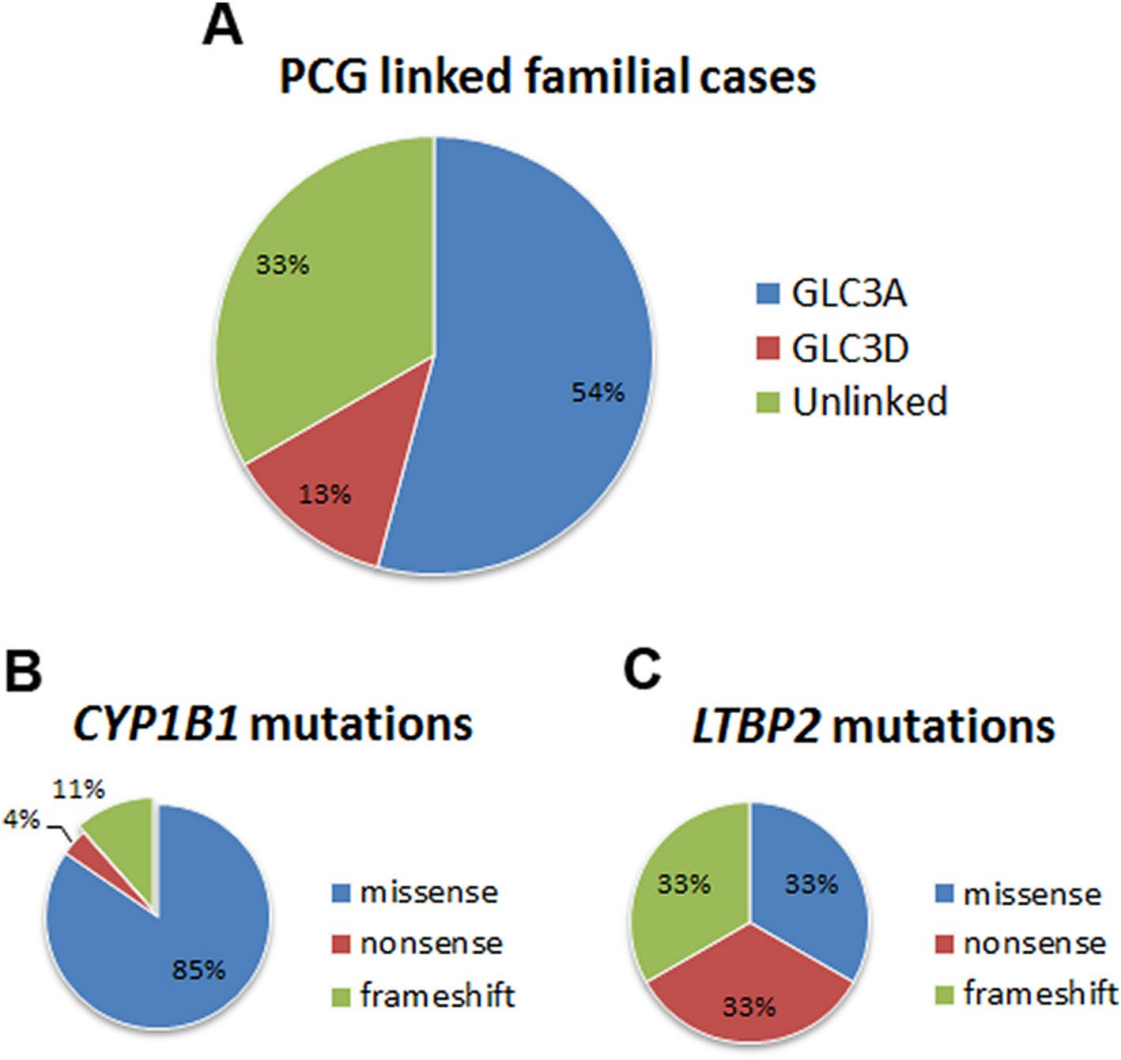
Pie chart illustrating the contributions of *CYP1B1* and *LTBP2* mutant alleles responsible for primary congenital glaucoma (PCG) in a cohort of familial cases of Pakistani descent. Distribution of (**A**) PCG loci, (**B**) *CYP1B1* mutations, and (**C**) *LTBP2* mutations in the PCG cohort. Missense, nonsense, and frameshift mutations were identified in both *CYP1B1* and *LTBP2*^20–22^.

**Figure 2:**
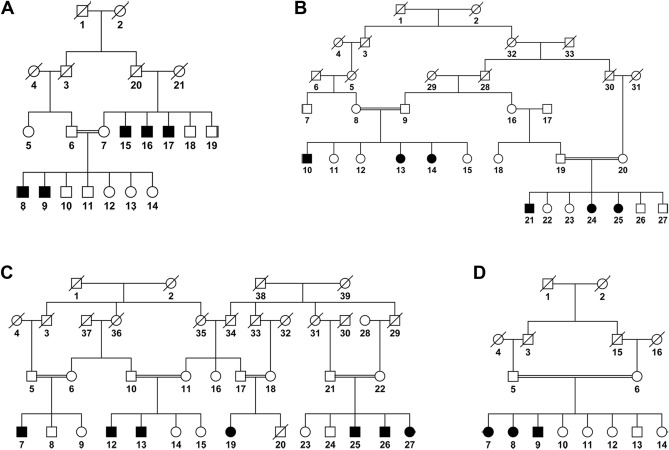
Pedigree drawings illustrating segregation of primary congenital glaucoma in four familial cases. (**A**) PKGL034, (**B**) PKGL036, (**C**) PKGL044, and (**D**) PKGL062 examined by exome sequencing. Squares are males, circles are females, filled symbols are affected individuals, a double line between individuals indicates consanguinity, and a diagonal line through a symbol is a deceased family member.

In an ongoing effort to identify the genetic determinants responsible for PCG in patients of Pakistani descent, we have ascertained a large cohort consisting of 48 families with at least two affected individuals per family. We previously employed short tandem repeat (STR) markers that localized two-thirds of our familial cohort (i.e. 32 families) to the reported PCG loci and as summarized in Fig. 1, sequencing identified mutations in *CYP1B1* and *LTBP2* in these families^20–22^. Importantly, the remaining one-third of the cohort (16 families) detailed in Table 1, excluded for linkage to reported PCG loci remains unsolved (Fig. 1). In the present study, we investigated the exomes of ten affected individuals manifesting cardinal systems of PCG from the four unlinked families (PKGL034, 036, 044 and 062) through next-generation sequencing (Fig. 2) along with four control samples consisting of genomic DNAs of affected individuals harboring mutations in *CYP1B1* and *LTBP2*. These four families were selected out of the 16 unlinked families based on a stronger pedigree structure with a higher number of affected individuals and consanguineous marriages within the family.

**Table 1:** Summary of the unlinked familial cases in our cohort with primary congenital glaucoma patients.

No	Pedigree ID	Total family members enrolled	Total affected individuals enrolled	Total affected individuals in the family	Exclusion analyses	Linkage to reported loci
1	PKGL011	10	4	4	Yes	Unlinked
2	PKGL017	13	2	2	Yes	Unlinked
3	PKGL018	6	2	2	Yes	Unlinked
4	PKGL023	7	2	2	Yes	Unlinked
5	PKGL024	8	2	2	Yes	Unlinked
6	PKGL027	10	5	6	Yes	Unlinked
7	PKGL029	5	2	2	Yes	Unlinked
8	PKGL034	10	3	5	Yes	Unlinked
9	PKGL036	7	3	6	Yes	Unlinked
10	PKGL044	15	7	7	Yes	Unlinked
11	PKGL052	5	2	2	Yes	Unlinked
12	PKGL055	11	5	6	Yes	Unlinked
13	PKGL056	11	2	2	Yes	Unlinked
14	PKGL061	18	6	6	Yes	Unlinked
15	PKGL062	7	3	3	Yes	Unlinked
16	PKGL064	7	2	3	No	Unlinked

Affected individuals in four families (PKGL034, 036, 044 and 062) underwent detailed medical examination including tonometry and slit-lamp microscopy at Layton Rahmatulla Benevolent Trust (LRBT) in Lahore, Pakistan. The ophthalmic examination in these four families revealed common symptoms of PCG including elevated IOP, increased corneal diameter, increased CD ratio, and visual acuity that was reduced to hand movement and/or light perceptions (Table 2). Moreover, bilateral buphthalmos, corneal opacity, central corneal haze, megalocornea, nystagmus, and myopic fundus were identified in some but not all affected individuals (Table 2).

**Table 2:** Clinical characteristics of primary congenital glaucoma patients.

Pedigree ID	Individual ID	Age at enrollment (years)	Visual acuity (OD/OS)	CD ratio (OD/OS)	IOP (OD/OS)	Corneal diameter
PKGL034	15	15	NPL/PL	1.0/NA	36/24^a^	Increased
	16	17	CF/CF	NA	NA	B/L > 14 mm
	17	23	PL/PL	NA	NA	NA
PKGL036	10	12	CF/CF	NA	37/23	NA
PKGL044	7	12	PL/PL	NV/NV	NA	Increased
	12	11	HM/HM	NV/NV	NA	Increased
PKGL062	7	5	CF/CF	NA	20^a^/32	Increased
	9	13	HM/HM	NV/NV	16^a^/14^a^	Increased

*CD ratio* cup to disc ratio, *CF* counting fingers, *IOP* intraocular pressure, *NPL* no light perception, *NV* no view, *OD* oculus dexter, *OS* oculus sinister, *PL* light perception, *HM* hand motion, *B/L* bilateral, *NA* not available

^a^IOP is controlled through surgery and/or medical treatment.

Prior to next-generation sequencing, we reconfirmed the exclusion of linkage to the reported PCG loci through STR marker-based exclusion analysis (Table 3). Once exclusion was reconfirmed, we selected 10 affected individuals from PKGL034, 036, 044 and 062, and performed whole exome sequencing as described in the materials and methods.

**Table 3:** Exclusion of GLC3A/*CYP1B1* (D2S2163, D2S177, D2S1346), GLC3B (D1S228, D1S402, D1S507, D1S2672), and GLC3D/*LTBP2* (D14S43, D14S1036, D14S61, D14S59, D14S74) through linkage analysis.

Pedigrees	Marker	0.0	0.01	0.05	0.09	0.1	0.2	0.3	0.4	Z_max_	ϴ_max_
PKGL034	D2S2163	0.38	0.39	0.42	0.43	0.42	0.36	0.25	0.12	0.43	0.09
	D2S177	− ∞	−1.64	−0.41	−0.06	−0.01	0.19	0.16	0.08	0.19	0.20
	D2S1346	− ∞	−1.45	−0.73	−0.51	−0.48	−0.33	−0.27	−0.16	−0.16	0.40
	D1S228	1.75	1.70	1.50	1.31	1.25	0.78	0.39	0.14	1.75	0.00
	D1S402	− ∞	−2.19	−0.88	−0.44	−0.38	0.00	0.09	0.09	0.09	0.30
	D1S507	−0.02	0.02	0.11	0.15	0.16	0.20	0.16	0.09	0.20	0.20
	D1S2672	− ∞	−4.50	−2.38	−1.63	−1.50	−0.72	−0.35	−0.14	−0.14	0.40
	D14S43	− ∞	−1.29	−0.56	−0.31	−0.27	−0.06	−0.01	0.00	0.00	0.40
	D14S1036	− ∞	−0.46	0.03	0.09	0.09	−0.05	−0.16	−0.13	0.09	0.09
	D14S61	− ∞	−1.50	−0.74	−0.47	−0.42	−0.16	−0.05	−0.01	−0.01	0.40
	D14S59	−2.90	−1.27	−0.61	−0.39	−0.35	−0.14	−0.05	−0.01	−0.01	0.40
	D14S74	− ∞	−2.96	−1.45	−0.90	−0.81	−0.28	−0.08	−0.01	−0.01	0.40
PKGL036	D2S2163	−2.23	−1.17	−0.55	−0.34	−0.31	−0.12	−0.05	−0.02	−0.02	0.40
	D2S177	− ∞	−1.75	−0.52	−0.18	−0.13	0.08	0.08	0.04	0.08	0.20
	D2S1346	− ∞	−3.32	−1.40	−0.78	−0.68	−0.15	−0.01	0.00	0.00	0.40
	D1S228	− ∞	−1.40	−0.20	0.10	0.15	0.26	0.17	0.06	0.26	0.20
	D1S402	− ∞	−3.26	−1.35	−0.74	−0.65	−0.14	−0.01	0.00	0.00	0.40
	D1S507	− ∞	−2.90	−1.53	−1.03	−0.94	−0.41	−0.16	−0.04	−0.04	0.40
	D1S2672	− ∞	−3.73	−1.78	−1.14	−1.03	−0.41	−0.15	−0.04	−0.04	0.40
	D14S43	− ∞	−0.58	0.00	0.14	0.15	0.17	0.11	0.05	0.17	0.20
	D14S1036	−2.23	−1.17	−0.55	−0.34	−0.31	−0.12	−0.05	−0.02	−0.02	0.40
	D14S61	− ∞	−5.13	−2.50	−1.61	−1.46	−0.59	−0.22	−0.06	−0.06	0.40
	D14S59	−2.23	−1.17	−0.55	−0.34	−0.31	−0.12	−0.05	−0.02	−0.02	0.40
	D14S74	− ∞	−2.62	−1.31	−0.86	−0.78	−0.33	−0.13	−0.04	−0.04	0.40
PKGL044	D2S2163	− ∞	−3.68	−1.72	−1.09	−0.98	−0.40	−0.18	−0.07	−0.07	0.40
	D2S177	− ∞	−3.39	−1.45	−0.84	−0.74	−0.22	−0.05	−0.01	−0.01	0.40
	D2S1346	− ∞	−2.16	−0.88	−0.49	−0.43	−0.13	−0.06	−0.04	−0.04	0.40
	D1S228	− ∞	0.55	1.03	1.07	1.06	0.80	0.44	0.14	1.07	0.09
	D1S402	− ∞	−1.73	−0.45	−0.06	−0.01	0.23	0.19	0.09	0.23	0.20
	D1S507	− ∞	−1.96	−0.70	−0.33	−0.27	−0.02	0.00	−0.02	0.00	0.30
	D1S2672	− ∞	−2.87	−0.98	−0.43	−0.34	0.06	0.11	0.06	0.11	0.30
	D14S43	− ∞	−5.29	−2.62	−1.71	−1.56	−0.65	−0.25	−0.07	−0.07	0.40
	D14S1036	− ∞	−3.11	−1.23	−0.67	−0.58	−0.16	−0.05	−0.02	−0.02	0.40
	D14S61	− ∞	−2.94	−1.05	−0.49	−0.40	−0.01	0.03	0.00	0.00	0.40
	D14S59	− ∞	−1.08	0.10	0.40	0.43	0.49	0.30	0.09	0.49	0.20
	D14S74	− ∞	−1.73	−0.45	−0.06	−0.01	0.23	0.19	0.09	0.23	0.20
PKGL062	D2S2163	−2.90	−1.27	−0.61	−0.39	−0.35	−0.14	−0.05	−0.01	−0.01	0.40
	D2S177	− ∞	−2.38	−1.08	−0.65	−0.58	−0.20	−0.06	−0.01	−0.01	0.40
	D2S1346	−3.16	−1.01	−0.38	−0.20	−0.17	−0.03	0.00	0.00	0.00	0.30
	D1S228	−2.76	−1.44	−0.80	−0.58	−0.55	−0.32	−0.21	−0.11	−0.11	0.40
	D1S402	−2.90	−1.27	−0.61	−0.39	−0.35	−0.14	−0.05	−0.01	−0.01	0.40
	D1S507	−1.71	−0.11	0.40	0.48	0.48	0.34	0.12	0.00	0.48	0.09
	D1S2672	− ∞	−2.72	−1.38	−0.91	−0.83	−0.35	−0.13	−0.03	−0.03	0.40
	D14S43	− ∞	−2.11	−0.87	−0.52	−0.46	−0.20	−0.13	−0.07	−0.07	0.40
	D14S1036	−2.46	−1.15	−0.54	−0.36	−0.33	−0.21	−0.18	−0.13	−0.13	0.40
	D14S61	− ∞	−2.11	−0.87	−0.52	−0.46	−0.20	−0.13	−0.07	−0.07	0.40
	D14S59	2.32	2.26	2.06	1.86	1.81	1.29	0.79	0.34	2.32	0.00
	D14S74	− ∞	−2.10	−0.87	−0.52	−0.46	−0.22	−0.16	−0.12	-0.12	0.40

− ∞ is negative infinity LOD score indicating recombination at the marker.

The quality control analysis of exome data revealed that > 99% of the reads were of 100 and 150 base pairs, while 95% of the sequencing data yielded a PHRED score, of 30 or above. High throughput sequencing yielded 39–71 million paired-end reads for each sample and ~ 39 to 69 million reads (> 97% of total reads) were uniquely mapped to the human genome (GRCh38.p13) representing an average of 89× to 127× coverage for all ten exomes (Table 4).

**Table 4:** Summary of the statistics of next-generation sequencing data.

Pedigrees	Individual ID	Total reads (10^6^)	Mapped reads (10^6^)	% of mapped reads	Sequenced bases (Mb)	Exome coverage (x)
PKGL034	8	61.90	60.40	97.58	6040.77	92.93
	9	55.27	55.10	99.69	8265.94	127.16
	17	70.89	69.02	97.36	6902.04	106.18
PKGL036	10	39.95	39.92	99.92	5988.56	92.12
	14	52.53	52.49	99.92	7873.50	121.13
PKGL044	13	59.34	58.09	97.89	5809.89	89.38
	19	62.28	61.01	97.94	6101.20	93.86
	26	71.55	69.79	97.53	6979.23	107.37
PKGL062	7	41.63	41.60	99.92	6240.00	96.00
	9	55.54	55.51	99.20	8265.00	127.15
PKGL015	8	57.85	57.81	99.93	8672.61	133.42
	13	59.05	59.00	99.92	8851.43	136.17
PKGL067	9	63.39	61.83	97.53	6183.43	95.12
	20	65.85	64.22	97.52	6422.02	98.80

**Figure 3:**
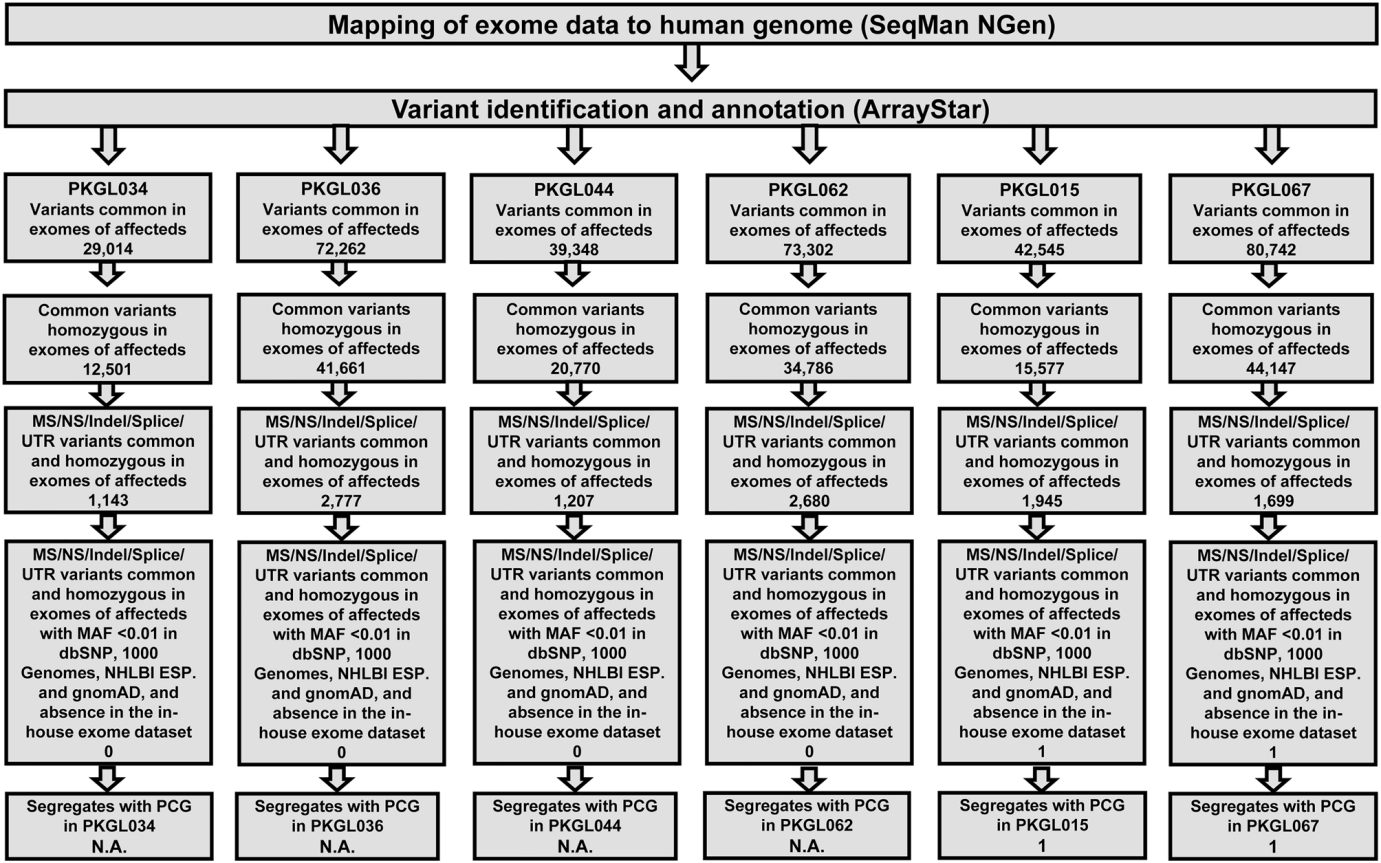
Flow chart depicting the protocol used for the bioinformatic analysis of whole exome sequencing data. The paired-end reads were aligned to the human genome (GRCh38.p13) using SeqMan NGen (Ver. 12; DNASTAR) and mapped reads were processed for variant calling and annotation with ArrayStar (Ver. 12; DNASTAR). The non-synonymous homozygous variants in the coding regions of the genome segregating in multiple affected individuals of the same family were selected for analyses. Any variants that did not adhere to MAF < 0.01 in public databases (i.e., dbSNP (Ver. 153), 1000 Genomes, NHLBI ESP, and gnomAD), and absent in the in-house exome dataset (> 50 ethnically matched exomes without PCG phenotype) were excluded from the analyses. *MS* missense, *NS* nonsense, *Indel* insertion/deletion, *UTR* untranslated region, *MAF* minor allele frequency, *N.A.* not applicable.

A multifaceted filtering approach was used for the identification of pathogenic variants responsible for the PCG (Fig. 3). Briefly, we included homozygous variants based on the disease segregation pattern (autosomal recessive) that were common in all affected individuals examined by exome sequencing. We interrogated missense and nonsense alleles, small insertions, and deletions (Indels), and variants at the splice-site and untranslated regions (UTRs) based on either their absence (novel) or MAF < 0.01 in public databases (i.e., dbSNP (Ver. 153), 1000 Genomes, NHLBI ESP, and gnomAD), and absence in the in-house exome dataset. Any variants passing the above-mentioned filtering criteria were examined for segregation with the disease phenotype in their respective families.

Whole exome sequencing identified 29,014 common variants in three affected individuals from PKGL034 (Fig. 3 and Supplementary Tables 1–3). We identified 1143 non-synonymous variants in coding, splice-site, and the UTRs (Fig. 3 and Supplementary Tables 1–3). As shown in Fig. 3, none of these 1143 variants passed the criteria of low allele frequency (MAF < 0.01). The exome sequencing identified 72,262 variants common to both affected individuals from PKGL036 (Fig. 3 and Supplementary Tables 4 and 5). We identified 2777 non-synonymous variants in coding, splice-site, and the UTRs (Fig. 3 and Supplementary Tables 4 and 5). As shown in Fig. 3, none of these 2777 variants passed the criteria of low allele frequency (MAF < 0.01). The exome sequencing identified 39,348 variants common to the three affected individuals from PKGL044 (Fig. 3 and Supplementary Tables 6–8). We identified 1207 non-synonymous variants in coding, splice-site, and the UTRs (Fig. 3 and Supplementary Tables 6–8). As shown in Fig. 3, none of these 1207 variants passed the criteria of low allele frequency (MAF < 0.01). Finally, whole exome sequencing identified 73,302 variants common in the two affected individuals from PKGL062 (Fig. 3 and Supplementary Tables 9 and 10). We identified 2680 non-synonymous variants in coding, splice-site, and the UTRs (Fig. 3 and Supplementary Tables 9 and 10). As shown in Fig. 3, none of these 2680 variants passed the criteria of low allele frequency (MAF < 0.01). Taken together, the whole exome analysis failed to identify any potential variants that would satisfy the criteria of causality including but not limited to low MAF.

To rule out the possibility that our next generation-based sequencing strategy is unable to identify causal mutations in genes responsible for PCG, we included two families, PKGL067, and PKGL015 that we previously reported to harbor mutations in *CYP1B1* and *LTBP2*, respectively^21,22^. We included two affected individuals from each family (individuals 9 and 20 of PKGL067, and individuals 8 and 13 of PKGL015; please see Refs.^21,22^ for pedigree drawings of PKGL067 and PKGL015, respectively) and performed whole exome sequencing as a positive control. Exome sequencing identified 80,742 variants common in the two affected individuals from PKGL067 (Fig. 3 and Supplementary Tables 11 and 12). We identified 1699 non-synonymous variants in coding, splice-site, and the untranslated region (Fig. 3 and Supplementary Tables 11 and 12). Importantly, we identified the missense allele c.1169G > A (p. Arg390His) in *CYP1B1* reported in PKGL067 responsible for PCG^21^. Likewise, exome sequencing identified 42,545 variants common in the two affected individuals from PKGL015 (Fig. 3 and Supplementary Tables 13 and 14). We identified 1945 non-synonymous variants in coding, splice-site, and the untranslated region (Fig. 3 and Supplementary Tables 13 and 14). Importantly, we identified the single base deletion (c.3427delC; p.Gln1143Argfs*35) in *LTBP2* reported in PKGL015 responsible for PCG^22^.

Although mutations in *CYP1B1* are the most common cause of PCG and are responsible for 27% of sporadic and 87% of familial cases worldwide^23^, a number of sporadic and familial PCG cases do not localize to *CYP1B1* (or to other reported PCG loci)^24^. Previously, two independent studies reported familial and sporadic cases of PCG that failed to identify pathogenic homozygous mutations through whole exome sequencing^24,25^. Kuchtey and colleagues^24^, presented results of exome sequencing using an autosomal recessive model of inheritance that failed to identify any causative variant in a familial case with six affected members. Sharafieh and colleagues^25^, performed whole exome sequencing of 24 families (30 PCG patients negative for mutations in both *CYP1B1* and *LTBP2*) but failed to detect any homozygous variants responsible for PCG in the affected cases.

It is worth noting that we have successfully applied linkage coupled with whole exome^26,27^, and whole genome^28,29^, sequencing approaches to delineate pathogenic variants responsible for ocular dystrophies. Likewise, a similar approach to delineate the genetic basis of extraocular diseases has been adopted by our group^30,31^, and many other groups^32,33^. Therefore, we propose genome-wide homozygosity or linkage mapping coupled with a whole genome sequencing approach to delineate the unknown genetic determinants of PCG in the 16 unsolved familial cases of PCG. Importantly, advancements in exome capture technologies i.e., to resolve the insufficient capture of GC-rich sequences and purging of other current limitations i.e., failure to detect large deletions or copy number variation (CNV) will also help to delineate the genetic basis of the unsolved familial cases in our cohort.

In summary, next-generation whole exome sequencing of multiple affected individuals from consanguineous families failed to identify the genetic basis of PCG. The lack of pathogenic variants in exome data strengthens the notion that compound heterozygous coding variants, non-coding RNA, or intronic variants in the inter- or intragenic regions are likely responsible for the PCG phenotype in the cohort of families excluded for mutations in *CYP1B1* and *LTBP2*.

## Materials and methods

### Subject recruitment and clinical evaluation

Patients affected with PCG were identified and recruited from the pediatric departments of LRBT Lahore. Informed written consent was obtained from all participating family members consistent with the tenets of the Declaration of Helsinki. This study was approved by the Institutional Review Board (IRB) of the Johns Hopkins University School of Medicine (Baltimore, MD), the National Institutes of Health (Bethesda MD), and the National Centre of Excellence in Molecular Biology (Lahore, Pakistan). The study was completed in accordance with the Declaration of Helsinki and all participating subjects provided informed consent before enrollment in the study.

A detailed medical and clinical history was obtained by interviewing members of the families. Ophthalmic examination including slit-lamp microscopy was performed at the LRBT Hospital. Elevated IOP > 16 mmHg for children and > 21 mmHg for adults, corneal edema, increased corneal diameter; > 12.0 mm and larger cup to disc (CD) ratio were inclusion criteria for the patients.

Approximately 10 ml of blood was drawn from all participating members and the samples were stored in 50 ml Sterilin Falcon tubes with 20 mM EDTA. Genomic DNA was extracted from white blood cells using a non-organic modified procedure as described^20–22^.

### Exclusion and linkage analysis

The reported loci/genes associated with PCG were screened by genotyping 12 polymorphic short tandem repeat (STR) markers spanning GLC3A/*CYP1B1* (D2S2163, D2S177, D2S1346), GLC3B (D1S228, D1S402, D1S507, D1S2672), and GLC3D/*LTBP2* (D14S43, D14S1036, D14S61, D14S59, D14S74). PCR amplification for genotyping was performed as described^20–22^. Two-point linkage analysis was performed using the FASTLINK version of MLINK from the LINKAGE Program Package^34,35^. The maximum two-point LOD scores were calculated using ILINK. PCG was analyzed as a fully penetrant autosomal recessive trait with an affected allele frequency of 0.001. The marker order and distances between respective markers were obtained from NCBI (National Center for Biotechnology Information; https://www.ncbi.nlm.nih.gov/) chromosomes 1, 2, and 14 sequence maps and Marshfield database (https://www.biostat.wisc.edu/~kbroman/publications/mfdmaps/).

### Next-generation whole exome sequencing

Whole exome library preparation and next-generation sequencing were performed in-house and commercially by Novogene Corporation Inc (Sacramento, CA). The exome libraries (in-house) were prepared using the Nextera Rapid Capture Expanded Exome kit (Catalog # FC-140-1005; Illumina Inc., San Diego, CA) according to the manufacturer’s protocol. Genomic DNA was quantitated using a Qubit Fluorometer (Qubit 2.0; Invitrogen, Carlsbad, CA). Approximately 50 ng of genomic DNA was subjected to an enzyme-based tagmentation process followed by amplification using barcode-specific indexes to prepare the genomic libraries. The genomic libraries were further processed for exome enrichment using expanded exome oligos (Illumina Inc., San Diego, CA). The exome-enriched libraries were quantitated using a high-sensitivity DNA chip on an Agilent 2100 Bioanalyzer (Agilent, Santa Clara, CA) and quantitative PCR (qPCR) according to the manufacturer's instructions. The bar-coded exome libraries were pooled and clustered using the TruSeq Cluster Kit (Ver. 3, Illumina, Inc. San Diego, CA) at 13 pM concentration and were paired-end (2 × 100 bp) sequenced on a single lane of HiSeq2000. The exomes (commercially) were captured by Agilent SureSelect Human All Exon kits (Ver.6) (Agilent Technologies, Inc. Santa Clara, CA) and sequenced in a paired-end fashion (2 × 150 bp) using the Illumina (Illumina Inc., San Diego, CA) HiSeq X-10 platform.

Lasergene Genomics Suite (DNASTAR, Madison, WI) was used for reference-guided genome alignment and variant calling/annotation of the whole exome sequencing data. The paired-end raw reads were aligned to the human genome (GRCh38.p13) using SeqMan NGen (Ver. 12) with default parameters. The mapped reads in the BAM file format were converted into the DNASTAR-specific format and processed for variant analysis. In the next step, mapped reads were further processed with ArrayStar (Ver. 12) for variant calling and annotation. The stringent criterion was used to filter false-positive results from the potentially causal variants. To ensure data quality, variants with low sequencing depth (< 2) and read quality (< Q20) were excluded.

Based on the disease segregation pattern (autosomal recessive) and consanguinity of familial cases, we assumed that a casual variant must be homozygous. We excluded all heterozygous variants from the analyses. Next, we removed all synonymous and intronic homozygous variants, and only non-synonymous homozygous variants located in the coding and splice regions of the genes were selected for further analysis. The non-synonymous homozygous variants were further scrutinized based on their absence (novel) or minor allele frequency (MAF) < 0.01 in public databases (i.e., dbSNP (Ver. 153), 1000 Genomes, NHLBI ESP, and gnomAD), and absence in the in-house exome dataset (> 50 ethnically matched exomes excluded for PCG). Note: Our strategy also includes the segregation analysis of potential causal variants with the PCG phenotype in their respective familial cases.

## Supplementary Information

Supplementary Legends.

Supplementary Table 1.

Supplementary Table 2.

Supplementary Table 3.

Supplementary Table 4.

Supplementary Table 5.

Supplementary Table 6.

Supplementary Table 7.

Supplementary Table 8.

Supplementary Table 9.

Supplementary Table 10.

Supplementary Table 11.

Supplementary Table 12.

Supplementary Table 13.

Supplementary Table 14.
