# Supplementary material for: Next-generation whole exome sequencing to delineate the genetic basis of primary congenital glaucoma
Source: Sci Rep. 2022 Oct 14;12:17218. doi: 10.1038/s41598-022-20939-5 (PMC9568502; doi:10.1038/s41598-022-20939-5)
Supplement: Supplementary file 1 — Supplementary Legends. [file 41598_2022_20939_MOESM1_ESM.pdf]

Next-generation whole exome sequencing to delineate the genetic basis of primary congenital glaucoma

Supplementary Table 1: Total variants identified in affected individual 08 of PKGL034.

Supplementary Table 2: Total variants identified in affected individual 09 of PKGL034.

Supplementary Table 3: Total variants identified in affected individual 17 of PKGL034.

Supplementary Table 4: Total variants identified in affected individual 10 of PKGL036.

Supplementary Table 5: Total variants identified in affected individual 14 of PKGL036.

Supplementary Table 6: Total variants identified in affected individual 13 of PKGL044.

Supplementary Table 7: Total variants identified in affected individual 19 of PKGL044.

Supplementary Table 8: Total variants identified in affected individual 26 of PKGL044.

Supplementary Table 9: Total variants identified in affected individual 07 of PKGL062.

Supplementary Table 10: Total variants identified in affected individual 09 of PKGL062.

Supplementary Table 11: Total variants identified in affected individual 09 of PKGL067.

Supplementary Table 12: Total variants identified in affected individual 20 of PKGL067.

Supplementary Table 13: Total variants identified in affected individual 08 of PKGL015.

Supplementary Table 14: Total variants identified in affected individual 13 of PKGL015.
